# From pituitary cells to prostate gland in health and disease: direct and indirect endocrine connections

**DOI:** 10.1007/s11154-025-09948-7

**Published:** 2025-02-06

**Authors:** André Sarmento-Cabral, Antonio C. Fuentes-Fayos, Fernando Mata Ordoñez, Antonio J. León-González, Antonio J. Martínez-Fuentes, Manuel D. Gahete, Raúl M. Luque

**Affiliations:** 1 Maimonides Biomedical Research Institute of Cordoba (IMIBIC), Avda. Menéndez Pidal s/n., Cordoba, 14004 Spain; 2 Department of Cell Biology, Physiology, and Immunology, University of Cordoba, Cordoba, 14014 Spain; 3 Reina Sofia University Hospital (HURS), Cordoba, 14004 Spain; 4 Department of Biomedical Sciences, Cedars-Sinai Medical Center, Los Angeles, CA 90048 USA; 5 Board of Governors Regenerative Medicine Institute, Cedars-Sinai Medical Center, Los Angeles, CA 90048 USA; 6 Faculty of Health Sciences, Alfonso X el Sabio University, Villanueva de la Cañada, 28691 Spain; 7 Department of Pharmacology, Faculty of Pharmacy, University of Seville, Seville, 41012 Spain; 8 CIBER Physiopathology of Obesity and Nutrition (CIBERobn), Cordoba, 14004 Spain

**Keywords:** Prostate, Pituitary, Hormones, Endocrine regulation, Prostate cancer, Obesity

## Abstract

The prostate gland is an endocrine-sensitive organ responding to multiple stimuli. Its development and function are regulated by multiple hormones (i.e. steroids such as androgens, estrogens and glucocorticoids) but also by other key hormonal systems such as those comprised by insulin-like growth factor 1 and insulin, which are sourced by different tissues [e.g. testicles/adrenal-gland/adipose-tissue/liver/pancreas, etc.). Particularly important for the endocrine control of prostatic pathophysiology and anatomy are hormones produced and/or secreted by different cell types of the pituitary gland [growth-hormone, luteinizing-hormone, follicle-stimulating hormone, and prolactin, oxytocin, arginine-vasopressin and melanocyte-stimulating hormone], which affect prostate gland function either directly or indirectly under physiological and pathophysiological conditions [e.g. metabolic dysregulation (e.g. obesity), and prostate transformations (e.g. prostate cancer)]. This review summarizes the impact of all pituitary hormone types on prostate gland under these diverse conditions including in vivo and in vitro studies.

## Introduction

**Fig. 1:**
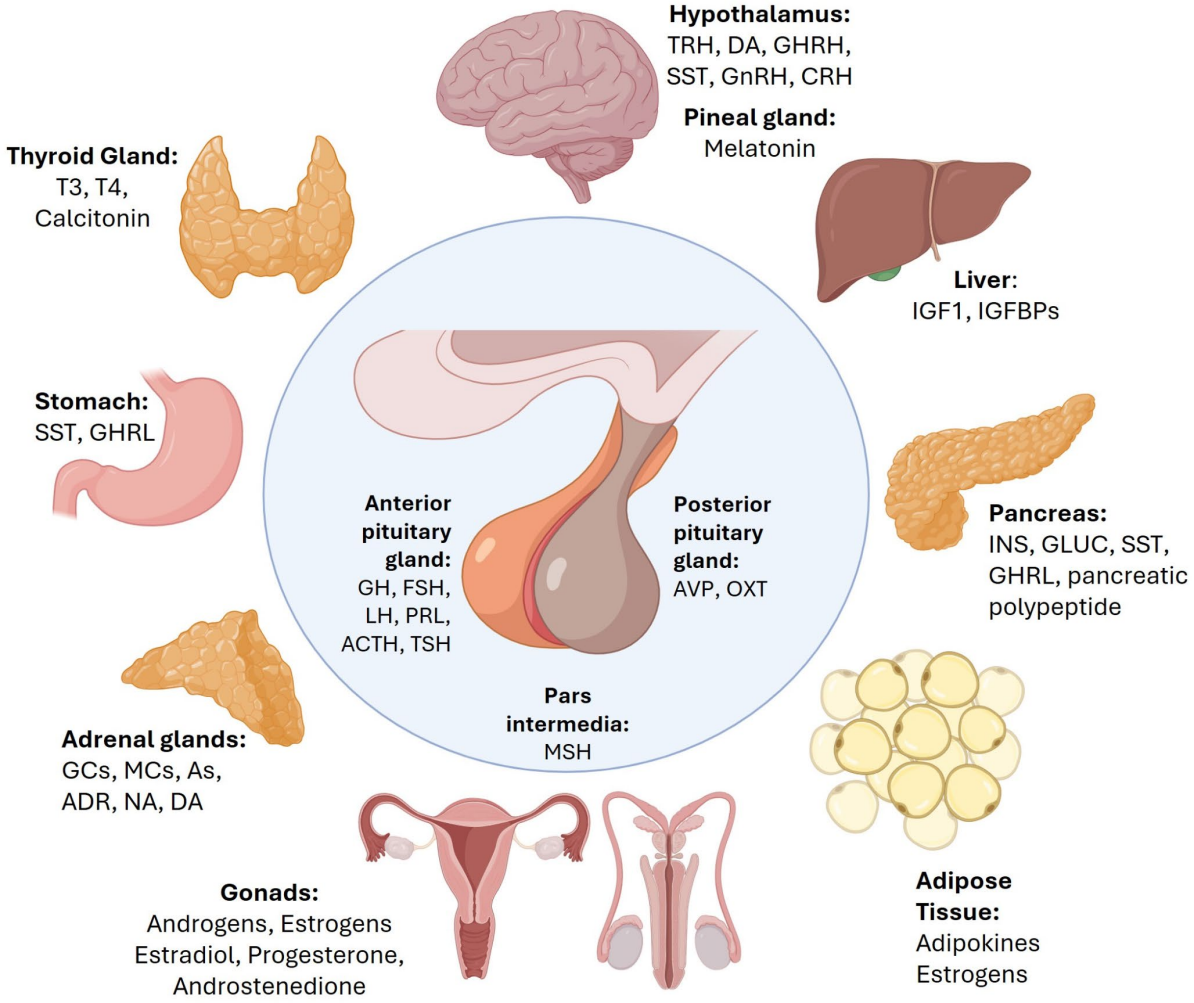
Main endocrine system comprised by glands and organs, which produce and/or secrete a wide variety of hormones. ACTH - Adrenocorticotropic Hormone; ADR - Adrenalin; As - Androgens, AVP - Arginine Vasopressin; CRH - Corticotropin Releasing Hormone; DA - Dopamine; FSH - Follicle Stimulating Hormone; GCs - Glucocorticoids; GH -Growth Hormone; GHRH - Growth Hormone Releasing Hormone; GHRL - Ghrelin; GLUC - Glucagon; GnRH - Gonadotropin Releasing Hormone; IGF1 - Insulin-Like Growth Factor 1; IGFBPs - Insulin-Like Growth Factor Biding Proteins; INS - Insulin; LH - Luteinizing Hormone; MCs - Mineralocorticoids; MSH - Melanocyte Stimulating Hormone; NA - Noradrenaline; OXT - Oxytocin; PRL - Prolactin; SST - Somatostatin; T3 - Triiodothyronine; T4 - Thyroxine; TRH - Thyrotropin Releasing Hormone; TSH - Thyroid Stimulating Hormone. This figure was created with BioRender.com and with Microsoft PowerPoint 365

The endocrine system is comprised by multiple glands and organs that produce and secrete a wide variety of regulatory hormones (Fig. 1), which play a critical role in regulating whole-body homeostasis and controlling the development and maintenance of almost all tissues and organs in the body. Specifically, the most relevant endocrine organs are the hypothalamus [that produces among others, thyrotropin-releasing hormone, dopamine, growth hormone (GH)-releasing hormone (GHRH), somatostatin, gonadotropin-releasing hormone (GnRH), and corticotropin-releasing hormone [1]], the pineal gland [that produces melatonin [2]], the thyroid gland [that produces triiodothyronine, thyroxine, calcitonin [3]], the pancreas [that produces insulin, glucagon, somatostatin, ghrelin, pancreatic polypeptide, etc [4]], the adrenal glands [that produces glucocorticoids, mineralocorticoids, androgens, adrenaline, noradrenaline, dopamine, etc [5]], the liver [insulin-like growth factor 1 (IGF1), Vitamin D and Angiotensinogen, etc [6]], the adipose tissue [that produces estrogens, multiple adipokines (e.g. leptin, adiponectin, resistin, etc.), etc [7]], the stomach [that secretes somatostatin, ghrelin, etc [8]] or the gonads [that secrete androgens, estrogens, etc [9]].

**Fig. 2:**
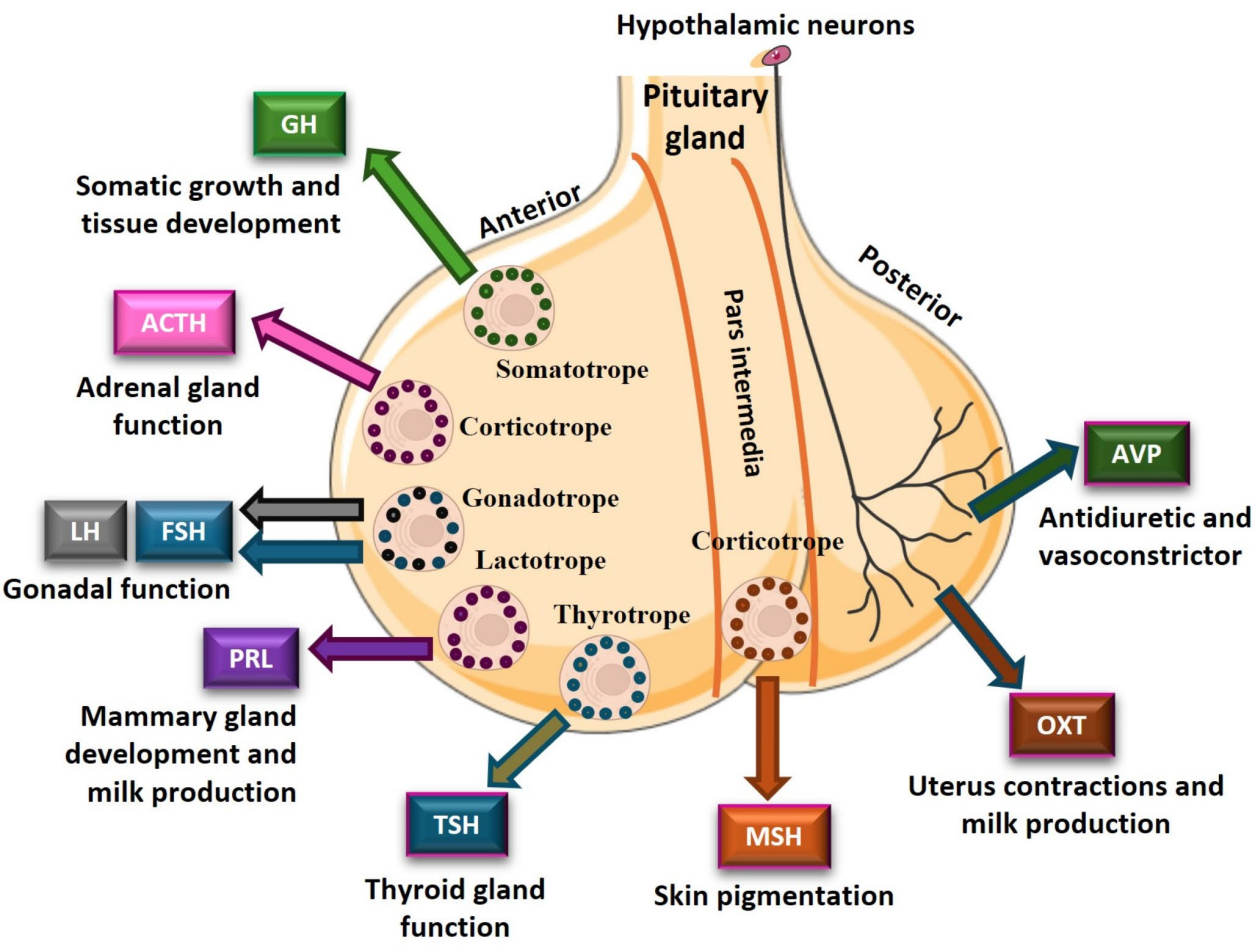
Representative model summarizing anatomy of pituitary gland and main (well-known) whole-body actions of the different hormones synthetized and/or secreted by the anterior and posterior pituitary gland. ACTH - Adrenocorticotropic Hormone; AVP - Arginine Vasopressin; FSH - Follicle Stimulating Hormone; GH -Growth Hormone; LH - Luteinizing Hormone; MSH - Melanocyte Stimulating Hormone; OXT - Oxytocin; PRL - Prolactin; TSH - Thyroid Stimulating Hormone. This figure was generated using Servier Medical Art, provided by Servier, licensed under a Creative Commons Attribution 3.0 unported license, with BioRender.com, and with Microsoft PowerPoint 365

However, among all endocrine tissues, the pituitary gland is considered as the “master gland” since it produces and secretes a series of key hormones that control the homeostasis and hormonal production of most endocrine organs and tissues [Fig. 2; i.e. GH, adrenocorticotropic hormone (ACTH), luteinizing hormone (LH), follicle-stimulating hormone (FSH), prolactin (PRL), thyroid stimulating hormone (TSH), melanocyte-stimulating hormone (MSH), oxytocin (OXT), and arginine vasopressin (AVP) [10]].

## The pituitary gland

The pituitary gland is a fundamental regulator of a plethora of relevant physiological functions such as growth, reproduction, puberty, lactation, metabolism, stress response, whole-body endocrine homeostasis, etc. This gland is located in a depression in the sphenoid bone, the *sella turcica*, at the base of the brain, and is comprised of the adenohypophysis (also known as anterior pituitary, which consists of the anterior lobe and intermediate, or pars intermedia) and the neurohypophysis (posterior lobe, or posterior pituitary). Anterior and posterior pituitary lobes are two distinct structures from the morphological and functional point of view, but exhibit a strong developmental and functional interplay [11] (Fig. 2).

The posterior pituitary gland is responsible for storing and releasing hormones produced by the hypothalamus [i.e. AVP, with actions on kidneys; and OXT, responsible for contractions of the uterus during labor] (Fig. 2). On the other hand, the anterior pituitary counts with five different cell types responsible for the synthesis and release of seven fundamental hormones, involved in the development and homeostasis of multiple tissues/organs as well as in the regulation of the plethora of relevant physiological functions (Fig. 2). Specifically: (i) somatotrope cells synthesize and secret GH, essential for somatic growth and tissue development, among other metabolic functions; (ii) gonadotrope cells are responsible for the synthesis and release of FSH and LH, important hormones for the regulation of the reproductive function and the proper prostate function; (iii) lactotroph cells synthesize and secret PRL, essential for mammary gland development and milk production during breastfeeding; (iv) corticotrope cells are the main responsible for the production and release of ACTH and MSH (the last one also secreted by the pars intermedia of the pituitary gland), which are crucial for the regulation of adrenal gland function and skin darkness, respectively; and, (v) finally, thyrotrope cells synthesize and secret TSH, responsible for the proper functioning of the thyroid [extensively reviewed in [12, 13]].

To finely regulate the production and secretion of these hormones and to appropriately exert all these functions, the pituitary gland receives, processes and integrates both central (mainly hypothalamic) and peripheral signals produced and secreted by numerous tissues and organs [10, 14]. These central and peripheral regulators signal through specific receptors on the pituitary gland, which comprehensively conveys this information to appropriately control the function of multiple key target organs [extensively reviewed in [12, 14–18]], including the prostate gland.

## Prostate gland as a component of the endocrine system

The prostate is an oval-shaped gland located between the bladder and the penis and with the size of a walnut (approximately 4 cm wide and 3 cm thick), which is part of the male reproductive system. This gland is responsible for the production and secretion into the urethra, during ejaculation, of a fluid that nourishes and protects the sperm [19, 20].

In the endocrine context, the prostate has been classically considered an exocrine gland that depends on other hormones (i.e. steroids) to maintain its size and normal secretory function. However, it is becoming evident that the prostate can also act as an endocrine, paracrine, and autocrine organ producing a variety of regulatory factors (i.e. hormones such as androgens and estrogens) that can influence the growth and function of the prostate itself, but also of other organs [21, 22].

**Fig. 3:**
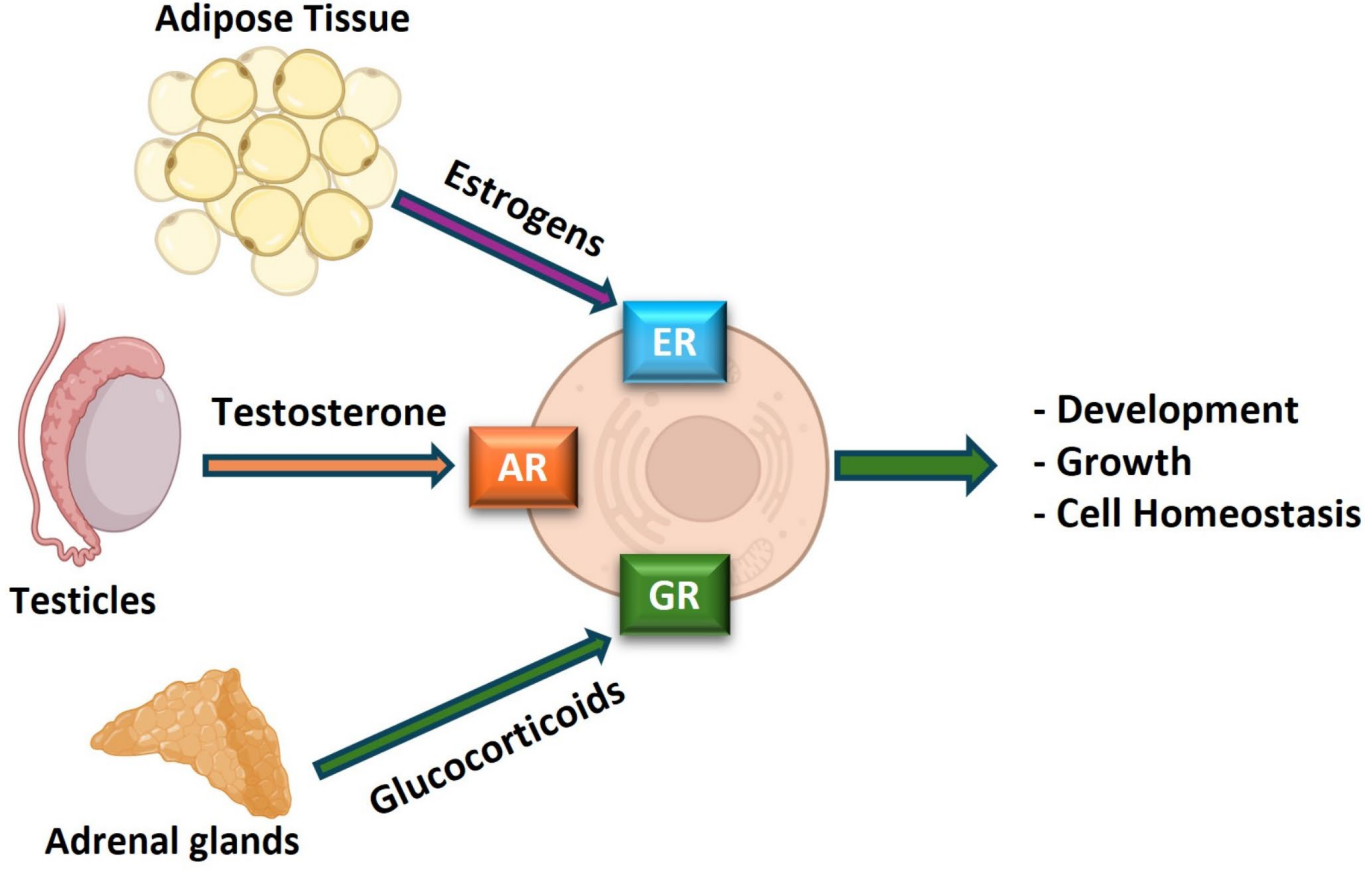
Direct regulation of prostate gland cells by hormones produced at the testicles (testosterone), adipose tissue (estrogens) and the adrenal gland (Glucocorticoids). AR - Androgen Receptor; ER - Estrogen Receptor; GR - Glucocorticoid Receptors. This figure was created with BioRender.com and with Microsoft PowerPoint 365

In fact, prostate gland development and function are regulated by multiple hormones [i.e. steroids hormones such as androgens, estrogens and glucocorticoids [19–21, 23, 24]] produced by different tissues [e.g. testicles, adrenal gland, adipose tissue, etc [25, 26]; Fig. 3]. In this regard, androgens are classically considered to be the most important hormones controlling prostate gland homeostasis, development, and function. The control of androgens production is highly complex since it is the result of a crosstalk between the hypothalamus, the pituitary gland, and the testicles (responsible for the production of 95% of the testosterone) [25, 27], but also with the participation of the adrenal glands, which, besides being the main source of glucocorticoids, also produce additional androgenic hormones as androstenedione and dehydroepiandrosterone [26]. In this sense, the majority of estrogens present in men are derived from the peripheral conversion, mainly in the adipose tissue, of androstenedione and testosterone to estrone and estradiol [28], however, estrogens have been also reported to be produced in the testicles [29]. As a result, androgens, estrogens, and glucocorticoids exert their actions at the prostate level via binding their cognate nuclear receptors (AR, ER and GR, respectively), thus regulating prostate function [29–32]. Hence, although the physiological role of glucocorticoids as regulators of prostatic biology is still unclear [33], the actions of androgens (mainly produced by the testicles) and estrogens (mainly produced in the adipose tissue) are crucial for prostate development, growth, and function [Fig. 3 [20, 34, 35]].

However, novel information from both human prostatic pathologies and animal models indicates that the endocrine control of prostatic pathophysiology is not as simple and straightforward as originally envisioned and might involve a wide variety of factors produced by other endocrine organs (e.g. adipose tissue, liver, pancreas, etc.) such as insulin, IGF1, different adipokines and microRNAs, and particularly from the pituitary gland, which also is a source of important direct or indirect regulators of the function of prostatic cells under both physiological and pathological conditions [19–21, 23, 24, 36–41].

## Understanding prostate changes and conditions

The prostate gland is about the size and shape of a walnut but, prostate enlargement is a very common condition associated with aging which can be accompanied by different prostate gland disorders [42]. In fact, prostate disorders are common, particularly in men aged over 50, and include inflammation (prostatitis), enlarged prostate [benign prostatic hyperplasia (BPH)], and prostate cancer (PCa). Despite prostatitis and BPH are non-tumoral pathologies, these conditions are associated with an increased risk to develop PCa [43], being this tumoral pathology the second most common cancer among male population worldwide [44]. In fact, PCa is highly dependent on androgen signaling, being androgen deprivation therapy the main pharmacological approach for treating PCa, inhibiting systemic androgen production. Although this hormonal therapy is especially effective when combined with radical therapeutic approaches such as radiotherapy, approximately 20% of the patients under this therapy will eventually develop resistance, progressing to a phenotype known as castration resistance prostate cancer (CRPC) [45–47], with limited therapeutic options.

The main accepted risk factors contributing to PCa are older age, ethnic origin, and genetic predisposition [44, 48, 49]; however, hormonal changes and endocrine disruptors [36, 44, 50–54], as well as oxidative stress, diet and obesity [37, 55, 56], have also been associated with PCa risk and aggressiveness. This plethora of risk factors and molecular alterations associated with PCa development may be related to the high intra-tumoral complexity and heterogeneity observed in PCa [57].

Nonetheless, as previously mentioned, the identification of a growing number of central and peripheral prostate gland regulators supports the view that the control of prostate function is more complex than originally envisioned. In this scenario, and as will be described below, there is growing evidence indicating that most of the pituitary hormones, secreted by the different pituitary cell types, can directly and indirectly influence the prostate gland homeostasis.

## Pituitary control of prostate gland under physiological and pathological conditions

**Fig. 4:**
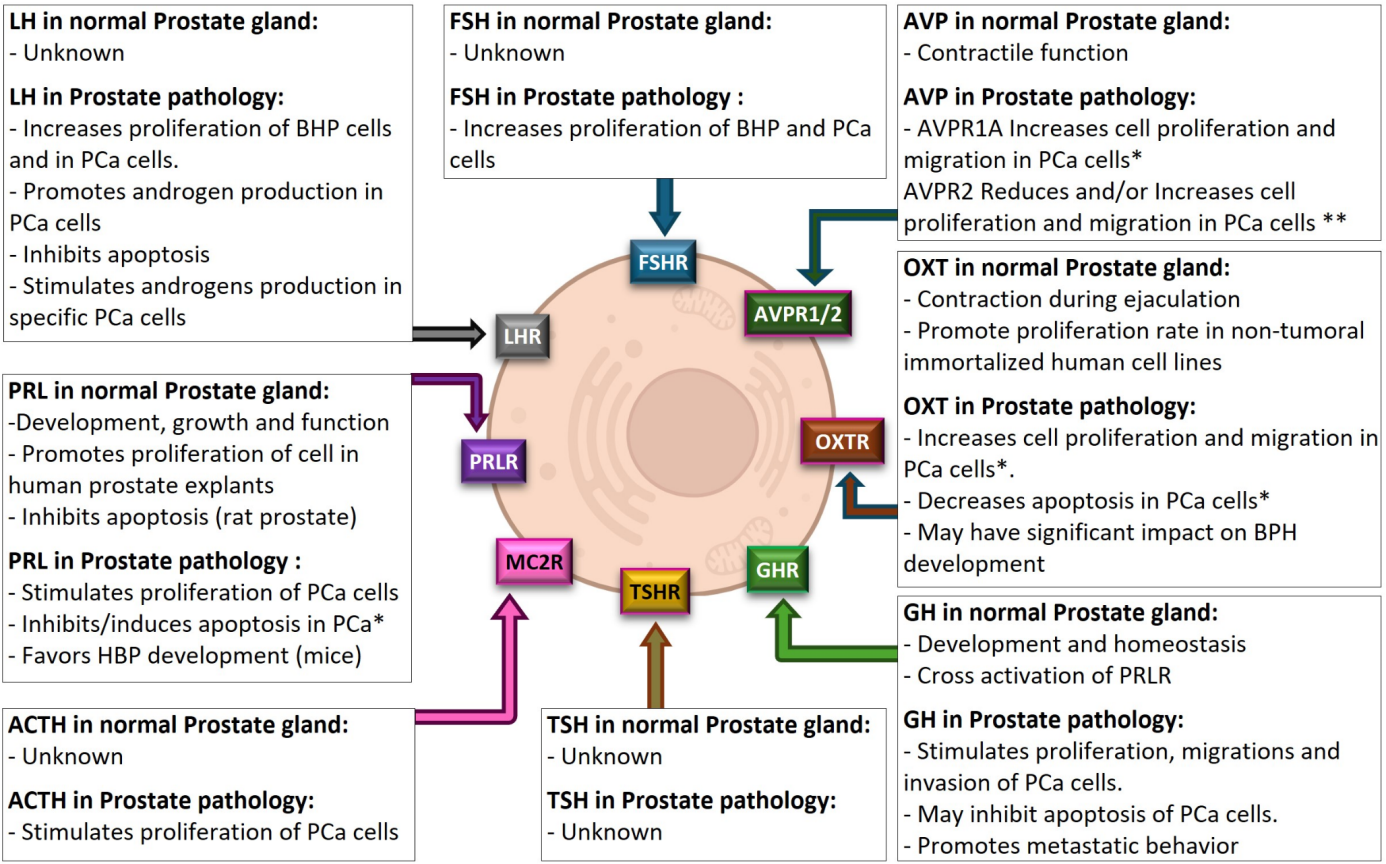
Summary of the DIRECT actions of different pituitary hormones on prostate gland. ACTH - Adrenocorticotropic Hormone; AVP - Arginine Vasopressin; AVPR - Arginine Vasopressin Receptor; FSH - Follicle Stimulating Hormone; FSHR - Follicle Stimulating Hormone Receptor; GH - Growth Hormone; GHR - Growth Hormone Receptor; LH - Luteinizing Hormone; LHR - Luteinizing Hormone Receptor; MC2R - Melanocortin Receptor 2; MSH - Melanocyte Stimulating Hormone; OXT - Oxytocin; OXTR - Oxytocin Receptor; PRL - Prolactin; PRLR - Prolactin Receptor; TSH - Thyroid Stimulating Hormone; TSHR - Thyroid Stimulating Hormone Receptor. BPH - Benigne Prostate Hyperplasia; PCa - Prostate cancer. This figure was created with BioRender.com and with Microsoft PowerPoint 365. * Cell line dependent, **Cell line dependent, contradictory results

**Fig. 5:**
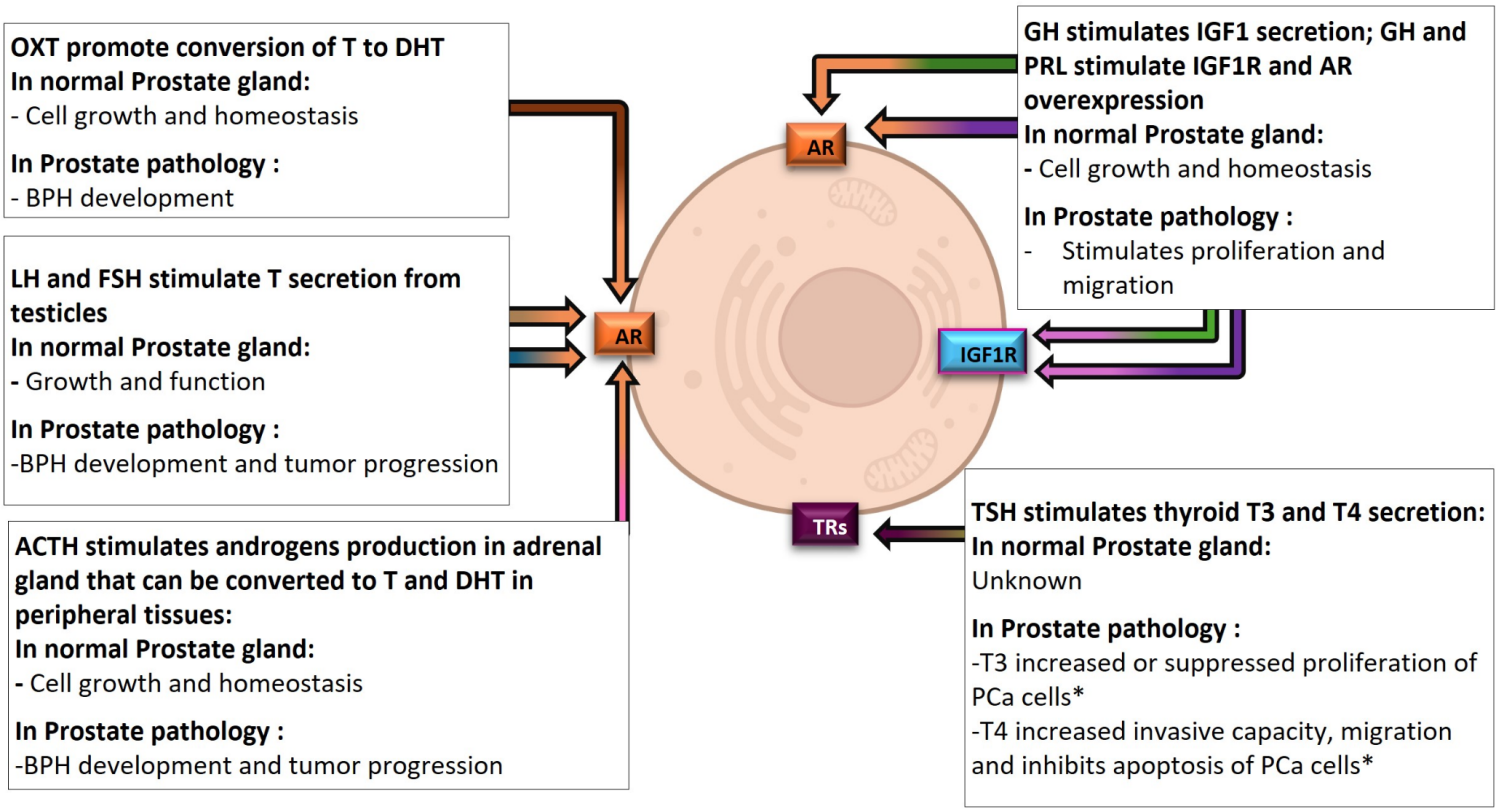
Summary of the INDIRECT actions of different pituitary hormones on prostate gland. ACTH - Adrenocorticotropic Hormone; AR - Androgen Receptor; BPH - Benign prostatic hyperplasia; DHT - Dihydrotestosterone; FSH - Follicle Stimulating Hormone; GH -Growth Hormone; IGF1 - Insulin-Like Growth Factor 1; IGF1R - Insulin-Like Growth Factor 1 Receptor; LH - Luteinizing Hormone; OXT - Oxytocin; PRL - Prolactin; T - Testosterone; T3 - Triiodothyronine; T4 - Thyroxine; TRs - Thyroid Hormone Receptors; TSH - Thyroid Stimulating Hormone; BPH - Benigne Prostate Hyperplasia; PCa - Prostate cancer. This figure was created with BioRender.com and with Microsoft PowerPoint 365. *cell line dependent

As mentioned above, the pituitary gland finely controls the physiology of different tissues through the secretion of multiple hormones, specially OXT and AVP from the posterior pituitary gland, and GH, FSH, LH, PRL, ACTH, MSH-α and TSH, produced and secreted from different cell types of the anterior pituitary gland. These hormones exert a direct action activating their specific receptors in the target tissues or may also present indirect effects through the modulation of other endocrine systems. This is the case of prostate gland wherein different pituitary hormones (OXT, AVP, LH, FSH, PRL, GH, ACTH, MSH and TSH) can exert direct (Fig. 4) or indirect (Fig. 5) actions as summarized below.

## Oxytocin

OXT is produced by the hypothalamus and released by the posterior pituitary gland [12]. Classically associated with uterus contraction and milk ejection, this hormone has also important actions on other organs through the activation of its specific receptor (OXTR) [58]. In fact, it has been documented that OXT acts on several male reproductive system tissues [58]. At the prostate level, a considerable amount of OXT mRNA has been detected [59]. In line with this, some reports have indicated that OXT could be important in the ejaculation process helping the contraction and tone of the prostate gland [60, 61]. Moreover, different OXT antagonists have recently been shown to reduce prostate contractility, which could be important for the premature ejaculation condition and to reduce involuntary contractions that may cause difficulties in urinating [61–64] (Fig. 4).

Notably, some studies have shown increased levels of OXT in plasma of PCa patients compared to controls without PCa [65], which might be involved in favoring the production of testosterone and its conversion to dihydrotestosterone (DHT) [60]. Thus, OXT could indirectly sensitize prostate gland cells to androgens since DHT has higher affinity for AR than testosterone [30], particularly in vitro this phenomenon was observed in LNCaP cells but not in PC-3 PCa cells [66]. Moreover, it has been shown that DHT and estrogens increased the OXT levels in primary BPH cell cultures promoting a positive feedback [67] (Fig. 5). Of note, immunohistochemistry studies in BPH tissues [68] and PCa samples [65] showed an increased expression of OXTR compared to normal prostate tissues. In this context, it has been suggested that OXT could have an important role in prostate gland growth and BPH development [69]. In fact, in vitro studies have shown that OXT treatment increased the proliferation rate of non-tumoral human prostate cell lines (RWPE and WPMY) [68, 70]. Accordingly, mice treated with OXT showed an enlargement of prostate gland when compared to non-treated control mice [70]. Controversially, another study reported no effects of OXT on normal human prostate epithelial cells [70]. Regarding tumoral behavior, treatment with OXT in vitro has been shown to increase the proliferation of LNCaP cells and decreased the apoptosis rates in both LNCaP and PC-3 PCa cells [65, 71]. Moreover, OXT promoted PC-3 cell migration, but did not impact this parameter on DU145 PCa cells [72] (Fig. 4).

## Arginine vasopressin

AVP, also known as antidiuretic hormone, is mainly produced by the hypothalamus and stored in the posterior pituitary gland. The actions of this hormone are mediated by three specific receptors [i.e. AVPR1A, AVPR1B and AVPR2 [73]], which are expressed in several tissues. Besides the well-known effects of AVP on blood pressure and water reabsorption in the kidneys, it has also been shown to impact many other mechanisms, including the modulation of pain perception, inflammation and cell proliferation, and also in metabolism and diabetes, [extensively reviewed in [73]]. However, with the exception of the contractile function [74] (Fig. 4), little is known about AVP actions on normal prostate function.

The expression of considerable amounts of AVPR1A mRNA has been recently reported in different PCa cell lines [75]. Specifically, expression of AVPR1A has been detected in 22Rv1, CWR-R1, C4-2B, and LNCaP-abl, but not in PC-3, DU145, LNCaP, nor in normal prostate-derived cells RWPE-1. Moreover, the knocking-out of this receptor resulted in decreased proliferation in castration resistant prostate cancer cell lines 22Rv1, CWR-R1, C4-2B, and LNCaP-abl, while overexpression of AVPR1A in LNCaP cells resulted in increased proliferation (in absence of androgen) and increased subcutaneous xenograft tumor growth in castrated mice. Furthermore, in vivo treatment with an AVPR1A antagonist (Relcovaptan) resulted effective in the control of orthotopic and subcutaneous xenograft tumors growth after mice castration, suggesting that the stimulation of this receptor promotes tumor growth in castration resistant PCa [75] (Fig. 4).

On the other side, the AVPR2 receptor is also expressed in PCa cells. Indeed, agonists of this receptor have been shown to reduce proliferation, doubling time and migration of PC-3 cells, indicating a potential antitumoral effect of the activation of this receptor [76]. Another study supported the same results showing that desmopressin treatment, a synthetic analog of AVP that binds to AVPR2, blocked proliferation in PC-3 and LNCaP cells, but only impacted the migration and invasion capacity of PC-3 cells, and prevented tumor growth in PC-3 xenografts [77] (Fig. 4).

Therefore, AVP seems to favor different tumoral behaviors depending on the activated receptor. Overall, these data could suggest an oncogenic role for the activation of AVPR1A, whereas an anti-tumoral effect is observed when AVPR2 is activated, and the expression ratio of these receptors may help to predict the evolutions of PCa. Contrary, a recent study presented contradictory results since showed that desmopressin could increase LNCaP, C4-2B and 22Rv1 cell proliferation, and the inhibition of AVPR2 (with Tolvaptan) and AVPR1A (with Relcovaptan), alone or combined, were able to reduce C4-2B xenograft tumors in mice [78] (Fig. 4).

## LH/FSH

It is widely accepted that the endocrine control of male reproductive system (i.e. testicles, prostate, etc.) is primarily regulated by the neuroendocrine activity of the hypothalamic-pituitary axis [17]. Specifically, LH and FSH are secreted in a pulsatile fashion and mediate their actions at the level of the testicles (i.e. production and secretion of testosterone) via specific transmembrane receptors [LHR (predominantly expressed in the interstitial Leydig cells) and FSHR (predominantly expressed in the Sertoli cells within the seminiferous cords/tubules), respectively]. Moreover, although testosterone produced from the testicles, in response to LH signaling, is one of the major regulators of prostate function [79] (Fig. 5), it has also been described that LHR and FSHR are also expressed at the prostate gland level [80], suggesting that both pituitary hormones might also act directly on the prostate gland, acting locally as hormones and growth factors.

As recently reviewed, LH and FSH have been associated with PCa [81]. Specifically, FSH was shown to stimulate proliferation in BPH primary cell cultures [80], being FSHR overexpressed in PCa compared to BPH [81]. In addition, serum FSH levels were positively associated with extraprostatic tumor development [82]. In the same line, a study proposed that exogenous FSH administration could increase xenograft tumors of androgen-independent human PC-3 PCa cells [in castrated mice with endogenous FSH suppression (degarelix-treated, a GnRH antagonist) or in castrated mice], and in androgen-independent human prostate cancer cells DU-145 (in degarelix treated mice) [83]. On the other hand, high baseline LH plasma levels were associated with worse PCa prognosis [84]. Indeed, LH was capable to stimulate proliferation and maintain androgen receptor expression in LNCaP and 22RV1 cells [85]. Furthermore, treatment with LH stimulated androgen production in LNCaP cells [85], and higher circulating levels of LH in humans were associated with increased Prostate Specific Antigen (PSA) levels and a worse prognosis [84] (Fig. 4).

Therefore, despite the well-known indirect effect of LH and FSH on prostate gland via androgens, limited but solid evidence is available supporting a direct effect of these two pituitary hormones on normal or tumoral prostate gland cells.

## PRL

PRL, secreted by lactotrope cells, is a well-known key player in lactation and mammary gland physiology in females. Additionally, PRL also participates in the normal development, growth and function of the prostate gland [86]. In fact, the discovery that human prostate expresses PRL-receptor (PRLR; a non-kinase single-pass transmembrane receptor) demonstrates that this organ might be a direct target of PRL [87]. Furthermore, PRL expression has also been demonstrated in the prostate itself, suggesting that this hormone might additionally act as a local growth factor via an autocrine or paracrine mechanism, distinct from its classical endocrine path [87]. Moreover, it has been demonstrated that PRL treatment directly stimulates cell proliferation in normal prostate explants organ cultures [87], and inhibits apoptosis of prostate epithelial cells in rats [88]. Interestingly, mice with overexpression of rat PRL, specifically on prostate gland (Pb-PRL mice), showed an enlargement of this gland, comparable to a BPH state [89, 90], supporting the autocrine importance of PRL in prostate (patho)physiology. Complementarily, the Pb-PRL transgenic mice with ubiquitous expression of PRL (Pb-PRL and Met-Δ1–9-G129R-hPRL double transgenic mouse model) presented a reduction in STAT5 activation and cell proliferation when compared with the prostate glands from Pb-PRL mice [90]. In fact, a potential prooncogenic role PRL in 22Rv1 and VCaP PCa cells has been suggested in vitro [91], indicating a putative connection between prostate tumorigenesis and excessive local PRL production. In this sense, it has been shown that the antiapoptotic and proliferative effects of PRL in PCa cells might be either direct (STAT5-mediated) [91, 92] (Fig. 4) or indirect [93] (Fig. 5), implying PRL-induced expression of receptors for growth factors such as IGF1R or AR [94]. However, these effects seem to be cell-line dependent since PRL induced apoptosis in LNCaP cells but had no effects on PC3 cells [95]. Nevertheless, the effects of PRL at the prostate gland level may be rather autocrine than paracrine since neither the PRL levels in plasma are not increased in PCa nor the PRLR is overexpressed in PCa samples compared to non-tumoral samples [86]. Despite the absence of differences of PRL (in plasma) or PRLR (in tissue) between PCa patients and healthy subjects, it was recently reported that metastatic CRPC patients with low PRL plasma levels had a better response to abiraterone treatment when compared to patients with high PRL plasma levels, suggesting a predictive value of PRL plasma levels of the response to abiraterone treatment [96].

Curiously, it has been proposed that prostate gland could be involved in the regulation of serum levels of different pituitary hormones. Specifically, subcutaneous administration of prostate extracts was able to restore PRL and FSH plasma levels of castrated and prostatectomized rats [97], which further suggests the presence of a direct regulatory feedback loop between the prostate and different pituitary cell types.

## GH

GH, secreted by the somatotropic cells in a pulsatile pattern, is a fundamental regulator of a plethora of relevant physiological functions such as growth, metabolism, whole-body endocrine homeostasis, reproduction, etc [98–100]. Moreover, GH and IGF1 [mainly secreted by the liver in response to GH [101]] system have been also shown to exert an important regulatory role in the development and homeostasis of prostate gland under normal and pathophysiological conditions [39, 40, 102, 103] (Figs. 4 and 5). Particularly, it has been demonstrated that prostate glands and human PCa cell lines (LNCaP, PC-3, MAT-Lu, MAT-LyLu, and Pif-1) express GH receptor (GHR) [37, 104]. Moreover, the transcription factor STAT5, activated by GH, has been shown to be upregulated in PCa promoting growth and metastatic behavior in vitro and in vivo [reviewed in [105]]. Another study reported that GH could promote proliferation, while pegvisomant (a GH antagonist) reduced apoptosis, regulated the expression of cancer-related genes in PTEN-P2, PTEN-CaP2, PTEN-P8, and PTEN-CaP8 cells [106], and increased LNCaP cells migration and invasion capacity [107] (Fig. 4).

In addition, GH was not only shown to be essential for prostate gland homeostasis, regulating its development [39, 40], but also for the local expression of IGF1 and its receptor (IGF1R) [94, 104, 108]. In this line, GH/IGF1 axis acts as an important regulatory system in prostate disorders and PCa development [37, 109–111]. In this sense, systemic and locally produced IGF1 can play an important role in prostate gland function regulating cell proliferation, differentiation and also apoptosis [37, 41, 106, 112], acting as a paracrine and/or autocrine factor. In fact, prostate gland and different PCa cell lines express both GHR and IGF1R [37]. In fact, IGF1 treatment was able to increase the proliferation in PC-3 and LNCaP cells and the migration rate in PC-3 cells [37] (Fig. 5). Interestingly enough, acromegaly in male patients, a disease characterized by hypersecretion of GH by the pituitary gland and as consequence increased plasma IGF1 levels, was associated with a 33% increased risk of the diagnosis of PCa [113] together with the incidence of different prostate conditions [reviewed in [103]].

Of note, human GH also has the capacity to activate PRLR [114], which signals through STAT5, suggesting that both GH and PRL could have a common participation in PCa development [88]. Interestingly, it was found that GH treatment stimulated AR synthesis at the prostate level, indicating that GH might act on this gland by potentiating the effects of androgens [94] (Fig. 5). On the other way around, androgens have been also shown to modulate GH synthesis and secretion in somatotroph cells [115], suggesting the existence of regulatory loop between the gonads and the somatotroph cells.

## ACTH and MSH

ACTH and MSH, secreted by corticotrope cells, are essential for the adrenal gland function and skin darkness, respectively [reviewed in [12, 13]]. Although ACTH and MSH exert different roles, both hormones are generated from the same mRNA transcript (proopiomelanocortin), suffering post-translational processing generating ACTH, α-MSH, β-MSH and γ-MSH (being α-MSH the most relevant among MSH products). These hormones exert their function activating the melanocortin receptors (MCRs1-5) with different affinities. Specifically, ACTH binds to MC1R and MC2R, whereas MSH binds to MC-1R, -3R, -4R and − 5R [13]. Despite the well-known roles of these hormones, there are limited and/or inconclusive studies addressing the role of ACTH and MSH on prostate gland function. Indeed, to the best of our knowledge, there are no studies testing MSH actions on prostate function or regulation. However, certain melanocortin receptors have been found to be expressed in prostate gland. Specifically, the ACTH receptor MCR2 is expressed at the prostate gland level, and ACTH treatment was found to increase the proliferation of PCa cell lines (LNCaP, PC-3 and DU145) [116] (Fig. 4). Additionally, it has been reported that ACTH can stimulate androgens secretion from adrenal gland, different from testosterone, which can be used as precursors and converted into testosterone in peripheric tissues [26] (Fig. 5). Interestingly, there are reports of a rare condition involving ectopic ACTH secretion by metastatic CRPC, with these patients presenting symptoms of Cushing’s syndrome [117–119], which could favor tumor progression in this conditions.

Therefore, despite some clues about the role (direct or indirect) of ACTH and MSH on prostate cells, the real impact is still unclear and further studies are needed to clarify the role of these hormones in prostate gland pathophysiology.

## TSH

TSH, secreted by thyrotrope cells, is crucial for the correct functioning of the thyroid gland [12], by activating its specific receptor (TSHR), stimulating the synthesis and secretion of the thyroid hormones Thyroxine (T4) and Triiodothyronine (T3) [3]. Interestingly, it has been observed that prostate gland shows TSH immunoreactivity and that PCa and normal prostate cells secrete TSH and/or thyrotropin-releasing hormone-like peptides [120, 121]. However, to our knowledge, to date no studies have been implemented to determine the direct effects of TSH on prostate cell function. A recent study showed an association of high levels of TSH with decreased risk of PCa [122, 123]. Although some studies evaluated the association between TSH and cancer incidence, the data obtained was not fully conclusive [reviewed in [124, 125]]. Notably, thyroid hormones Thyroxine (T4) and Triiodothyronine (T3), have been shown to exert different effects on PCa depending on the hormone and cell model studied [124] (Fig. 5). Specifically, T3 increased the proliferation rate in LNCaP cells, but not in PC-3 cells [126]. On the other hand, in vivo studies using LNCaP xenograft tumors showed that T3 reduces tumor growth [127]. In the same cell line, T4 hormone increased the invasive capacity of LNCaP cells [127], and promoted migration and repressed detachment-induced apoptosis of PC-3 cells [128]. Interestingly, DU145 cells were irresponsive to both T3 and T4 hormones [127] (Fig. 5). Therefore, the direct impact of TSH is still unclear and further studies are needed to clarify its specific role in prostate gland pathophysiology.

## Connection between pituitary cells and prostate gland in obesity

Obesity (OB) is a complex metabolic disease that impacts million people worldwide [129]. This disease is characterized by profound alterations in the homeostasis of the organism affecting multiple endocrine systems, and consequently favoring the development of different pathologies, including different cancer types [130]. Indeed, lifestyle has been estimated to cause at least 40% of cancers, wherein 4–8% may be attributed to overweight [131], suggesting a significant pathophysiological association between OB and various cancer types, including PCa [51, 132–134]. In this context, alterations in the secretion pattern of different pituitary hormones under OB conditions have been described [135], wherein different and complex hypothalamic-pituitary regulatory circuits might be directly or indirectly involved in the pathophysiological relationship between OB and prostate gland dysregulations and/or transformations.

For instance, plasma GH levels are reduced in OB probably due to the hyperinsulinism state [136–138], a pathophysiological condition that could promote the reduction of IGF binding proteins, followed by an increase in free IGF1 availability [136, 139], wherein all these systemic alterations might subsequently impact prostate gland pathophysiology [37, 38, 140], Likewise, plasma prolactin levels have also been reported to be altered in OB [i.e. generally elevated with weight gain [141]], wherein hyperprolactinemia might directly influence prostate gland function at multiple levels [142]. In fact, preclinical and epidemiological studies as well as analyses of human tissues strongly support the contribution of PRLR signaling to prostate gland physiology under normal or altered metabolic conditions [143, 144]. In terms of corticotrope cells, although the available data regarding ACTH levels in OB is still controversial [135, 141], it has been reported that an enhanced ACTH response to AVP, and reduced cortisol clearance, could influence prostate gland pathophysiology via MC2R or GRs [116, 145, 146]. Similarly, TSH levels are often found to be slightly elevated in OB [147], with normal or slightly elevated free T3 and T4 levels [135], wherein elevated TSH levels in OB may be due to the ability of leptin to stimulate thyrotropin-releasing hormone expression and synthesis, and subsequently TSH release [141]. Thus, this complex regulatory axis involving changes in TSH, T3/T4 and leptin levels might in conjunction also influence prostate gland pathophysiology under OB conditions. In terms of the gonadotroph axis, it has been demonstrated that OB and/or diabetes, through both central (hypothalamic) and peripheral mechanisms, typically result in low serum testosterone levels, while alterations in plasma LH and FSH levels in response to OB are more controversial (i.e. found to be in the normal range or reduced) [141, 148]. Therefore, since PCa is highly dependent on androgens at the initial stages, alterations in testosterone levels in OB have been proposed to directly influence prostate pathophysiology [149, 150]. Interestingly enough, it has also been reported that OB itself, independently of the testosterone levels was associated with high grade and metastatic PCa [151].

Finally, in terms of posterior pituitary hormones, while limited information is available about AVP levels in OB, a report suggested that AVP levels may be increased in OB since its surrogate marker copeptin was found to be positively correlated with body mass index [152], wherein this increased AVP levels could potentially influence prostate pathophysiology *via* activation of AVPRs. Additionally, plasma OXT levels are reported to be consistently decreased in OB and several other conditions of abnormal glucose homeostasis [153], suggesting that dysfunction of the oxytocin system could underlie the pathogenesis of multiple organs expressing OXTR in OB [154], including the prostate gland under health and disease (e.g. PCa) conditions [58, 60, 65, 155].

## Clinical implications of pituitary hormones dysregulation on prostate pathologies

Based on the information summarized in previous sections, it would be plausible to suggest that changes in circulating levels of different pituitary hormones may potentially have relevant clinical implications for the management of different prostate pathologies, including BPH and PCa under normal and altered metabolic conditions (e.g. OB). In particular, the dysregulation of the hypothalamic-pituitary-gonadal (HPG) axis, characterized by changes in LH and FSH, directly affects gonadal androgen production, a critical driver of prostate growth and malignancy [79]. In fact, the suppression of LH *via* GnRH agonists or antagonists is fundamental for an androgen deprivation therapy (ADT), a basis treatment for advanced PCa [156]. However, compensatory elevations in FSH following ADT may contribute to disease progression, highlighting the need for additional therapeutic molecular targets. In line with this and also related to androgens signaling, prolactin dysregulation may increase androgen receptor (AR) signaling and tumor growth [94], posing to the prolactin antagonists as potential adjunct therapy. Furthermore, androgens produced by adrenal gland under ACTH signaling [157] may play an important role in CRPC, where ADT resistance becomes the main mechanism of PCa progression [158]. In fact, some drugs (e.g. abiraterone) inhibit adrenal steroidogenesis by targeting this pathway [158]. Furthermore, as previously mentioned, alterations in GH/IGF1 axis have been associated with increased PCa aggressiveness, wherein targeting of this axis (e.g. using IGF-IR targeted therapies) showed promising anti-tumor effects [159, 160].

Additionally, the clinical implications of pituitary hormone alterations in the management of prostate pathologies become even more complex in the context of OB. In fact, OB-related leptin resistance and elevated inflammatory cytokines may amplify prolactin secretion [141], enhancing AR signaling and tumor progression [94]. Moreover, altered ACTH dynamics in OB [145] may also contribute to increased adrenal androgen production, potentially supporting androgen synthesis in CRPC. Furthermore, OB is associated with suppressed GH and altered IGF1 activity [136, 137, 139], which may influence cellular proliferation and contribute to aggressive prostate pathology. These interactions highlight the importance of weight/metabolic control as adjuvant strategies in the management of prostate disease, as they may modulate pituitary hormonal axes and improve therapeutic outcomes.

In addition, in the last years it has been shown that different epigenetic modification such as DNA methylation, histone acetylation, regulation of methyltransferases and demethylases are implicated in OB and cancer [161, 162], including PCa development and heterogeneity [163–166]. Specifically, these alterations modulate gene expression patterns of prostate cells, affecting cellular proliferation and differentiation, which may increase the susceptibility to the development of BPH and/or PCa. In this context, current evidences suggest that alterations in multiple hormonal systems may induce epigenetic modifications, potentially altering AR activity and its downstream signaling networks [167]. However, to the best of our knowledge, there is no available information about the potential effects that different pituitary hormones could be exerting on epigenetic modifications at the prostate gland level. This information could offer valuable insights into the intricate relationship between the pituitary and prostate gland in both health and disease, especially under OB conditions, as it may reveal new opportunities for developing therapeutic interventions and identifying biomarkers.

## Concluding remarks

According to the classical view, the endocrine control of prostatic physiology and anatomy was thought to be mostly mediated through testosterone levels produced by the testicles and controlled by a specific hypothalamic-pituitary axis [18]. However, over the last years, it has become evident that the endocrine control of prostatic pathophysiology is far more complex than originally envisioned. In fact, it involves a wide variety of factors produced by other endocrine organs (e.g. adipose tissue, liver, pancreas, etc.), and particularly other hormones from the pituitary gland, that may act either directly on the prostate gland (summarized in Fig. 4), or indirectly via activation of crucial prostate gland regulators (summarized in Fig. 5). Finally, it must also be considered that prostate gland can act as an endocrine, paracrine, and autocrine tissue producing a variety of regulatory factors (i.e. hormones such as androgens and estrogens) that can influence the growth and function of the prostate itself, but also of other endocrine tissues [21, 22]. In this scenario, although evidence has been reported, given the inconclusive data and/or cell line-dependent actions of many of the referred pituitary hormones on the prostate gland, further studies should be implemented to elucidate the precise effects, the underlying mechanisms of action, and the network of interactions of these endocrine regulators, under normal conditions and pathophysiological states (e.g. OB and/or PCa).

In fact, the current understanding of pituitary hormone alterations remains constrained by limited insights into the complex regulatory mechanisms controlling their secretion, feedback loops, and downstream effects under health and disease conditions. While significant steps have been made in delineating their physiological roles, the complexity of hormone interactions (in normal and under OB), receptor signaling, and tissue-specific responses continues to pose challenges. Therefore, integrating hormonal and metabolic interventions could optimize PCa treatments efficacy.

## Data Availability

No datasets were generated or analysed during the current study.

## Abbreviations

ACTH: Adrenocorticotropic Hormone
AR: Androgen Receptor
AVP: Arginine Vasopressin
AVPR1A: Arginine Vasopressin Receptor 1A
AVPR1B: Arginine Vasopressin Receptor 1B
AVPR2: Arginine Vasopressin Receptor 2
BPH: Benign Prostatic Hyperplasia
CRPC: Castration Resistance Prostate Cancer
DHT: Dihydrotestosterone
ER: Estrogen Receptor
FSH: Follicle-Stimulating Hormone
FSHR: Follicle-Stimulating Hormone Receptor
GH: Growth Hormone
GHR: Growth Hormone Receptor
GHRH: Growth Hormone (GH)-Releasing Hormone
GnRH: Gonadotropin-Releasing Hormone
GR: Glucocorticoids Receptor
IGF1: Insulin-Like Growth Factor 1
LH: Luteinizing Hormone
LHR: Luteinizing Hormone Receptor
MCR1: Melanocortin Receptor 1
MCR2: Melanocortin Receptor 2
MCR3: Melanocortin Receptor 3
MCR4: Melanocortin Receptor 4
MCR5: Melanocortin Receptor 5
MSH: Melanocyte-Stimulating Hormone
OXT: Oxytocin
OXTR: Oxytocin Receptor
PCa: Prostate Cancer
PRL: Prolactin
PRLR: Prolactin Receptor
STAT5: Signal Transducer and Activator of Transcription 5
T3: Triiodothyronine
T4: Thyroxine
TSH: Thyroid Stimulating Hormone
TSHR: Thyroid Stimulating Hormone Receptor
